# Thornton’s Dose-Book and Prescription Writing

**Published:** 1902-07

**Authors:** 


THORNTON'S DOSE-BOOK AND PRESCRIPTION WRITING.—Dose-book and Manual of prescription writing: with a list of the official drugs and preparations, and the more important newer remedies. By E. Q. Thornton, M. D., demonstrator of Therapeutics, Jefferson Medical College, Philadelphia. Second edition, revised and enlarged. Octavo, 362 pages, illustrated. Philadelphia and London: W. B. Saunders & Co., 1901. Bound in flexible leather, $2 net.

In every respect this little work is deserving of the highest praise. It contains in condensed and easily accessible form a vast amount of information absolutely indispensable to both advanced students and practicing physicians. Every one of its 360 or more pages may be studied with advantage. In dealing with official preparations, methods of prescribing, drugs and their preparation, etc., all unnecessary matter has been eliminated, thus simplifying greatly the study of these all-important subjects. R.

